# An unusual case of tuberculous pericarditis diagnosed exclusively by positive acid-fast staining

**DOI:** 10.1590/0037-8682-0084-2021

**Published:** 2021-04-28

**Authors:** Diego Chemello, Luís Felipe Teixeira Neumaier, Ivy Bauer Lovatel

**Affiliations:** 1 Universidade Federal de Santa Maria, Departamento de Clínica Médica, Santa Maria, RS, Brasil.; 2 Universidade Federal de Santa Maria, Programa de Pós-graduação em Ciências da Saúde, Santa Maria, RS, Brasil.

A 70-year-old woman was admitted to the hospital because of shortness of breath. The symptoms started one month before admission and worsened over the following weeks. On the day before admission, the patient’s condition worsened, and she experienced substernal chest pain and palpitations. She had a history of hypertension and type 2 diabetes mellitus.

On physical examination, mild distress, tachypnea, and tachycardia were observed. There was a pulsus paradoxus of 10 mmHg (110 mmHg decreasing to 100 mmHg). She was normoxemic while breathing ambient air. Mild jugular venous distention was also observed. Heart auscultation revealed mildly diminished heart sounds.

**FIGURE 1:**
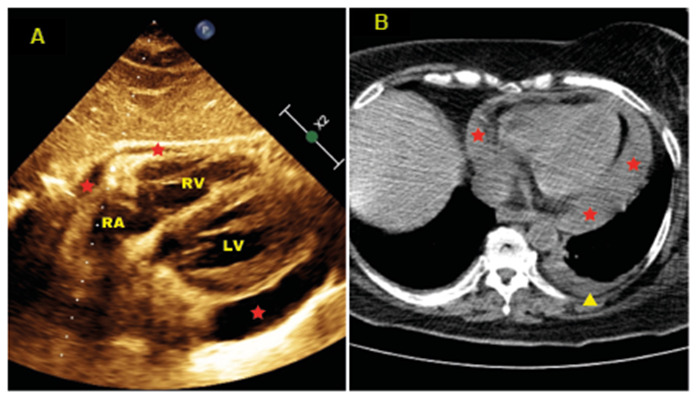
**(A)** - Bedside echocardiographic image (long parasternal axis view) showing signs of pericardial effusion (red *). **(B)** - Chest computed tomography showing circumferential pericardial effusion (red *) and mild left pleural effusion (yellow arrowhead). **RA:** right atrium; **RV:** right ventricle; **LV:** left ventricle.

A 12-lead electrocardiogram revealed sinus tachycardia. Bedside echocardiogram showed circumferential pericardial effusion and respiratory variation in the intracardiac flows; all findings were consistent with increased intrapericardial pressure (Figure 1A). Chest computed tomography (CT) showed mild left pleural effusion and signs of circumferential pericardial effusion (Figure 1B).

**FIGURE 2:**
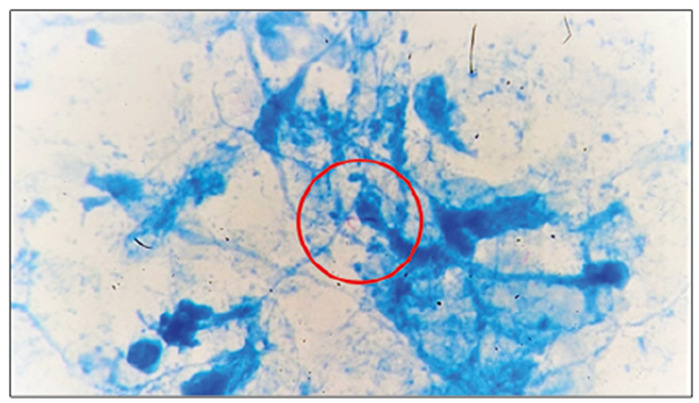
Tuberculous bacilli (acid-fast bacilli, red circle) present within the pericardium infiltrate.

The patient underwent an urgent surgical pericardial window with a pericardial biopsy. Approximately 550 mL of bloodstained fluid with exudative properties was removed from the pericardium. Fluid analysis revealed three leukocytes. The adenosine deaminase level was 37 IU/mL. Polymerase chain reaction for *Mycobacterium tuberculosis* complex showed negative results. Histological analysis of the pericardium did not reveal any granulomas; however, acid-fast bacilli were identified (Figure 2). Therefore, the diagnosis of tuberculous pericarditis was confirmed, and quadruple therapy was started according to the guidelines^1^.

The patient had a favorable evolution and was discharged after one week. Posterior culture from the pericardial tissue was negative.
